# Cabergoline plus metformin therapy effects on menstrual irregularity and androgen system in polycystic ovary syndrome women with hyperprolactinemia

**Published:** 2015-02

**Authors:** Azam Ghaneei, Akram Jowkar, Mohammad Reza Hasani Ghavam, Mohammad Ebrahim Ghaneei

**Affiliations:** 1 *Department of Endocrinology, Shahid Sadoughi Hospital, Shahid Sadoughi University of Medical Sciences, Yazd, Iran.*; 2 *Shahid Sadoughi University of Medical Sciences, Yazd, Iran.*; 3 *Khatam-Ol- Anbia Clinic, Yazd, Iran.*

**Keywords:** *Polycystic ovarian syndrome*, *Cabergoline*, *Metformin*, *Menstrualcycle*, *Laboratory findings*

## Abstract

**Background:**

30% of patients with polycystic ovary syndrome (PCOS) show mild, transient hyperprolactinemia. It is suggested that a reduction of the dopamine inhibitory effect might raise both prolactin and luteinizing hormone.

**Objective:** To investigate the adjuvant cabergoline therapy effects on menstrual irregularity and androgen system in PCOS women with hyperprolactinemia.

**Materials and Methods:**

This randomized clinical trial was done on 110 polycystic ovary syndrome women with increased serum prolactin concentration [1.5 fold more than normal level (>37.5 ng/ml)]. Participants were divided into two groups: Case group (n=55) treated with metformin 1gr/day and cabergoline 0.5 mg/week for 4 months and control group (n=55) treated with metformin 1g/day and placebo weekly. Testosterone, prolactin, and dehydroepiandrosterone sulfate level were measured before and four months after intervention in two groups. Also, situation of menstrual cycles asked and recorded before and after intervention.

**Results:**

We found decrease in the mean of dehydroepiandrosterone sulfate, weight and total testosterone level in the two groups after intervention but their changes were not significant. Patients in case group showed a significant decrease in serum prolactin level before and after intervention (p<0.001), but no difference was found in control group. All patients in both studied groups had irregular menstrual cycles, which regulate after intervention and the difference was significant (p=0.02).

**Conclusion:**

The results showed that cabergoline can be used as a safe administration in PCOS patients with hyperprolactinemia to improve the menstrual cycles. Considering that the administration of cabergoline plus metformin may reduce the required duration and dose of metformin, patient acceptability of this approach is higher.

## Introduction

Polycystic ovary syndrome (PCOS) is a common reproductive and endocrinopathy disorder that is the most common cause of an ovulatory infertility. It has a wide spectrum of clinical findings including: hyperandrogenemia, hyperinsulinemia, increasing luteinizing hormone (LH) secretion, menstrual irregularity, hirsutism, and infertility (1-10). According to the Rotterdam criteria PCOS is characterized by a combination of oligo/ amenorrhoea, clinical or endocrine signs of hyperandrogenemia and polycystic ovaries (11). PCOS involves ~5-10% of women in reproductive age which affect not only on their fertility but also on their health (6). In fact, menstrual irregularity exists in approximately all obese and 72% of thin PCOS patients. Although producing the oocyte is more in PCOS patients, but the produced oocytes have the lower quality which causes the infertility disorders and increases abortion rate (12, 13). 30% of patients with PCOS show also modest rise in prolactin levels (14-17). Increasing of serum prolactin in these patients can be found in both follicular and luteal phase of the normal and stimulated cycles (18). 

Prolactin is a secreted hormone by lactotrop cells of the anterior pituitary gland. Milk production, reducing reproductive and sexual function can induce as the effects of this hormone (19). Laboratory examination showed that the increase in prolactin can cause reduction in the ovulation and the number of ovarian follicles. These findings are resulting from the luteolytic function of this hormone, although, it’s mechanism is not clear (20). In human, released dopamine by the hypothalamus is the most important inhibitor of prolactin secretion (21). 

Although there are different ideas but studies have shown that central dopaminergic mechanisms are also effective on contribution of the gonadotropins and LH secretion. This reducing effect of dopaminergic mechanisms can cause abnormal secretion of prolactin and LH, this disorder in PCOS patients is seen with increased levels of prolactin (22, 23). Nowadays, Metformin (N, N-dimethylimidodicarbonimidic diamide) is used to control steroid related disorders and improve the menstrual irregularity and infertility in PCOS women (24, 25). 

Cabergoline is a dopamine receptor agonist that its formula is C_26_H_37_N_5_O_2_. Cabergoline is more effective in patients with hyperprolactinemia in comparison with bromocriptin (26, 27). Cabergoline has higher affinity than dopamine D2 receptors and has the serum half-life of 43-hour limit. So this drug with such effects can be helpful in the treatment of hyperprolactinemia patients (28, 29). Few studies have been performed about using long-acting dopamine agonists with inhibiting of prolactin (cabergolin) in patients with PCOS (30, 31). 

For example, Ajossa *et al* reported that chronic administration of cabergoline increases uterine perfusion in PCOS patients (28). Other study showed that cabergoline in PCOS patients can provide better ovarian response, reduced the risk of ovarian hyperstimulation syndrome (OHSS), and decreased serum prolactin concentration with no increase in pregnancy rate (29). Some researches indicate that the administration of cabergoline can normalize androgen levels and improve the menstrual irregularity in women with PCOS. They conclude that cabergoline by decreasing prolactin secretion can play a useful role in treatment of menstrual irregularities in PCOS patients (30-31).

This study was performed to investigate the effects of cabergoline therapy in addition to metformin (usual treatment) in PCOS patients on androgen system, hormone modification and menstrual irregularity.

## Materials and methods

This randomized clinical trial was done on 110 PCOS women with increased serum prolactin concentration [1.5 fold more than normal level (>37.5 ng/ml)], who referred to endocrinology clinic of Khatamal Anbiaa Cilinic, Shahid Sadoughi University of Medical Sciences, Yazd, Iran. The study protocol was approved by the ethics committee of the Shahid Sadoughi University of Medical Sciences. Informed consent was obtained from all participants.

Our participants were selected from patients with clinical symptoms of PCOS such as hirsutism, obesity, menstrual irregularity (mainly oligomenorreha) based on the Androgen Excess Society standards (AES). All participants were examined for other causes of increased prolactin levels such as TSH test, pituitary magnetic resonance imaging (MRI) for detection of prolactineoma and other disorders or tumors that can increase prolactin, and then women who had diagnosed as PCOS according to the latest AES standards enrolled in the study. Patients who had other endocrine disorders (such as thyroid disorders), history of cardiovascular disease, history of using the increasing prolactin drugs, women who wanted to be pregnant, and who couldn’t tolerate the cabergoline were excluded from the study. 

Demographic characteristics including age, history of drug usage, and the menstrual situation were asked. One fasting venous blood sample (5 ml) was taken from each participant. Serum testosterone, prolactin and dehydroepiandrosterone sulfate (DHEAS) level was measured with ELISA (enzyme-linked radio immune sorbent assay) method before treatment. Then, the patients were allocated into two groups by the table of random numbers: 1) oral administration of 1 gr/day metformin and 0.5 gr cabergoline weekly for 4 months as case group (n=55), and 2) oral administration of 1 gr/day metformin with placebo every week for 4 months as the control group (n=55). Testosterone, prolactin and DHEAS level were again measured four months after intervention in two groups. Also, situation of menstrual cycles was asked again and recorded.

**Statistical analysis**

Statistical analysis was carried out using SPSS software (Statistical Package for Social Sciences, version 18.0, SPSS Inc, Chicago, Illinois, USA) and chi-square, Mann-Whitney, Wilcoxon, and Student’s *t* test were used to detect significant differences between two groups. The level of significance was set at p<0.05.

## Results

**Figure 1:**
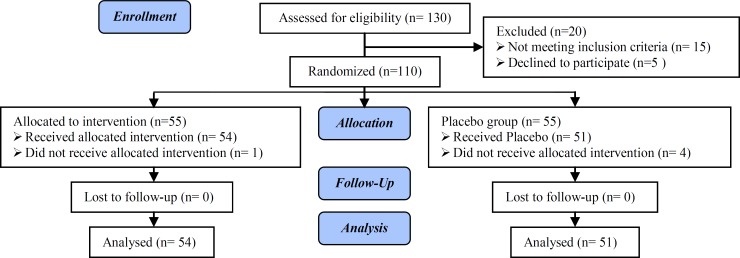
Consort Flow Diagram

In the present study 110 patients were studied into two groups of case (n=55) and control group (n=55) (Figure 1). One of the participants from case and four women from the control group were excluded of the study because of not referring. Finally, 54 patients in case group and 51 patients in the control group were analyzed. Both groups were matched in age and body mass index (BMI) (Table I). In our analysis, the mean of prolactin, DHEAS, and weight in case group before and after intervention were different significantly (p<0.001). In control group, the mean of prolactin was not significantly different before and after intervention. But, there was statistically significant difference between mean of DHEAS and weight (p<0.001). According to Wilcoxon test mean of testosterone in two groups before and after intervention was different significantly (p=0.04) (Table II). 

There were no statistically significant differences between two groups before and after intervention regarding to mean of DHEAS (p=0.09), weight (p=0.73), and level of testosterone (p=0.07) changes. But the mean of prolactin changes was different significantly (p<0.001) (Table III). All patients in both studied groups had irregular menstrual cycles, which regulate after intervention and the difference was significant. The rate of regulation after intervention in case group was 58.2% and in control group was 36.4% (p=0.02) (Table IV).

**Table I:** Baseline characteristics in case and control groups

**Variables**	**Case group**	**Control group**	**p-value**
Age (year)	25.20 ±4.8	25.16 ±4.66	0.97*
BMI (kg/m^2^)	29.70 ± 2.52	29.50 ± 2.54	0.66*
Testosterone (nmol/L)	1.02 ± 0.02	0.93 ± 0.15	0.009
DHEAS (μmol/L)	401.25 ± 12.57	410.72 ± 75.72	0.62*
Prolactin (IU/L)	41.98 ± 2.52	46.45 ± 35.99	0.36

DHEAS: Dehydroepiandrosterone Sulfate

Mann-Whitney test

* Student’s *t* test

**Table II:** Comparison of prolactin, DHEAS, weight, and testosterone before and after intervention in case and control groups

**Time**	**Prolactin**		**DHEAS**		**Weight**		**Testosterone**	
	**Case**	**Control**	**Case**	**Control**	**Case**	**Control**	**Case**	**Control**
Before intervention	42.05 ± 6.11	46.96 ± 37.33	402.76 ± 121.18	411.88 ± 77.85	73.18 ± 7.64	72.64 ± 8.51	4.38 ± 24.94	2.26 ± 9.85
After intervention	7.11 ± 5.24	38.21 ± 8.06	231.68 ± 121.49	291.29 ± 109.42	72.2 ± 8.44	71.05 ± 9.12	2.06 ± 8.72	0.87 ± 0.22
p-value	>0.001*	0.08*	>0.001*	>0.001*	>0.001	>0.001	>0.001*	0.04*

Data presented as mean ± standard deviation

DHEAS: Dehydroepiandrosterone Sulfate

Paired-sample t test

* Wilcoxon test

**Table III:** Comparison of prolactin, DHEAS, weight, and testosterone changes in case and control groups before and after intervention

Groups	**DHEAS**	**Weight**	**Testosterone**	**Prolactin**
Case	171.08 ± 153.52	0.98 ± 2.72	2.32 ± 0.294	34.94 ± 7.70
Control	120.59 ± 116.22	1.59 ± 6.93	1.39 ± 0.26	8.75 ± 8.50
p-value	0.09*	0.73*	0.072*	<0.001*

* mann-whitney test

**Table IV:** Menstrual irregularities in case and control groups

	**Case group **	**Control group**	**p-value**
Regular menstrual cycle	32 (58.2%)	19 (36.4%)	0.02
Irregular menstrual cycle	22 (41.8%)	32 (63.6%)	

Numbers are presented as number (%).

Chi-square test

## Discussion

This study was designed to evaluate the effect of cabergoline on menstrual irregularity and the level of serum androgens (testosterone, DHEAS and prolactin) in PCOS women with mild hyperprolactinemia. The mean of total testosterone, DHEAS, and weight in two groups after intervention were reduced significantly, but their changes were not different significantly. The mean of prolactin was reduced significantly in case group but was not significantly different before and after intervention. 

Also, according to our results, the Use of cabergoline plus metformin was effective in improving irregular menstrual cycles, by reducing the required duration and dose of metformin. Patient acceptability of this approach is higher and therefore it is more effective. Although, the mechanism is not clear, many authors have demonstrated an inhibiting role of dopamine and its’ agonists on LH secretion and androgen concentration both in normal and hyperprolactinemia women. Dopamine agonist was proposed as a useful tool in the management of PCOS (26, 27). 

But in our study, the androgen changes were not significantly difference in two groups. The results of Paoletti and colleagues showed that the use of cabergoline 0.5 mg/week for 4 months in the treatment of PCOS patients can cause a decrease in the levels of LH and improve irregular menstrual cycles, but the recovery rate was not mentioned (30). Also, Prelevic *et al* showed that the compounds of L-DOPA and bromocriptin (dopamine agonist) in PCOS patients with hyperprolactinemia can cause a significant decrease in LH levels in comparison to the normoprolacinemia group (32). As a result, we expect to increase the number of ovulatory cycles and a regular menstrual cycle. This result was obtained in our study. Ajossa *et al* also found that PCOS patients have increased vascular resistance and the use of cabergoline causes a significant increase in uterine blood supply in PCOS patients (28). No similar study on combination of metformin therapy and cabergoline has been reported.

As expected, the mean of prolactin in the case group treated with metformin and cabergoline decreased after intervention, and the difference was significant before and after intervention. While in treated group by placebo and metformin there was no significant difference before and after intervention, which it is justified according to the effect of cabergoline in reducing the level of prolactin, as noted in the previous studies, such as Papaleo *et al* and Prelevic *et al *studies (29, 32). Prelevic *et al* showed a significant relation between basal levels of prolactin and the changes in prolactin level due to the inhibitory effect of bromocriptin and L DOPA. They suggested that the reduction in the inhibitory effect of dopamine in the hypothalamus can be a reason for inappropriate increased levels of LH and prolactin in PCOS patients with hyperprolactinemia (32).

In our study all of patients had menstrual irregularity, that in 58.2% of women treated with cabergoline and metformin and in 36.4% of metformin and placebo treated group the menstrual cycles became regular which this difference was significant in two groups. Kriplani *et al* surveyed 66 PCOS patients with menstrual cyclicity which their irregularity improved after 6 month’s treatment by metformin (33). 

Also kedikvo *et al* reported that menstrual irregularity was improved by metformin therapy (850 mg twice a day) in PCOS patients, which in contrast to our results, the dose of metformin and duration of treatment were more (34). In addition, Prelevic *et al* detected that L DOPA compounds and bromocriptin in PCOS patients with hyperprolactinemia can cause a significant difference in their LH level, increase ovulation cycles, and finally regulate their menstrual cycles (32). Ajossa *et al* showed using cabergoline can increase the uterine perfusion in PCOS patients (28). These studies confirm our results, although a similar study which used the combination of metformin and cabergoline in PCOS patients was not found. 

Finally, based on the evidence of dopamine agonists efficacy in reducing LH, prolactin, vascular resistance and increased uterine blood supply to the uterus, it can be concluded that the use of cabergoline with metformin, improve irregularity of menstrual cycles more than use of metformin alone. According to the results of mentioned studies, in PCOS patients for more effectiveness of metformin, we need to use higher dose and long term treatment, that many of these patients are not able to tolerate. Whereas by combination of metformin with cabergoline as a safe and weekly used drug, and also use lower dose of metformin we can reach our aim over a shorter time period.

In our study, the mean of testosterone before and after intervention was significantly different in both group, but testosterone changes was not significantly different between two groups. In similar study was done by Singh *et al* the mean of total testosterone before and after metformin therapy was not significantly different (35). Velija *et al* reported that treatment by metformin showed significant reduction in testosterone level in PCOS patients (36). Also, Kazerooni *et al* in their study detected a significant difference in total testosterone level in PCOS patients (37). Results of other study showed that testosterone rate and DHEAS significantly reduced after treatment by dopamine agonists (38) which coordinated with our study. 

In PCOS patients DHEAS level can be high which is seen in approximately 50% of patients without ovulation; adrenal is the source of this hormone (39). Banaszewska *et al* showed metformin therapy cause no significant different in DHEAS level in these patients, but cause a reduction in total testosterone (40). Similar results were also detected in another study by the same procedures (41). A similar study didn’t find significant difference in DHEAS rate after treatment by metformin, which is not in correlation with our study (42). Also, Kenneth *et al* showed that using bromocriptin can cause significant difference in DHEAS and prolactin level in PCOS patients, and suggested that prolactin may be involved in producing adrenal androgens (43). Totally, results of our study and similar studies demonstrated that using dopamine agonists such as cabergoline have better effect on reducing DHEAS in comparison with using metformin alone (21, 43, 44).

## Conclusion

Our results showed that cabergoline can be used as a safe administration in PCOS patients with mild hyperprolactinemia to improve the menstrual irregularity. Use of cabergoline plus metformin is effective in improving irregular menstrual cycles and may reduce the required duration and dose of metformin. Patient acceptability of this approach is higher and therefore it is more effective.

## Note

***This article extracted from M.D. thesis. (Akram Jowkar)***

***Registration ID in IRCT: IRCT2014121420086N2***
